# Vision and Hearing Difficulties and Life Expectancy Without ADL/IADL Limitations: Evidence From the English Longitudinal Study of Ageing and the Health and Retirement Study

**DOI:** 10.1093/gerona/glad136

**Published:** 2023-05-26

**Authors:** Paola Zaninotto, Asri Maharani, Giorgio Di Gessa

**Affiliations:** Department of Epidemiology and Public Health, University College London, London, UK; Department of Nursing, Faculty of Health and Education, Manchester Metropolitan University, Manchester, UK; Department of Epidemiology and Public Health, University College London, London, UK

**Keywords:** Activities of Daily Living, Dual sensory difficulty, Hearing, Life expectancy, Vision

## Abstract

**Background:**

Hearing and vision difficulties are some of the most common deficits experienced by older adults. Having either visual or hearing difficulties increases the risk of comorbidity, disability, and poor quality of life. So far, however, few studies have examined the association between vision and hearing difficulties on life expectancy without activities of daily living (ADL) or instrumental ADL (IADL) limitations (LEWL).

**Methods:**

Data came from the English Longitudinal Study of Ageing and the Health and Retirement Study in the United States from 2002 to 2013. The outcome was defined as reporting 2+ limitations with ADL/IADL. Life expectancy was estimated by discrete-time multistate life table models for hearing and vision difficulties separately as well as for combined vision and hearing difficulties by sex and age.

**Results:**

Thirteen percent of men in England and the United States had ADL/IADL limitations, whereas, for women, it was 16% and 19% in England and the United States. At all ages, either vision or hearing difficulty was associated with shorter LEWL compared to no difficulties. Dual sensory difficulty (vision and hearing) reduced LEWL by up to 12 years in both countries. At the ages of 50 and 60 in England, hearing difficulty was associated with fewer years lived without ADL/IADL limitations than vision difficulty. In contrast, in the United States, vision difficulty led to fewer years lived without ADL/IADL limitations than hearing difficulty.

**Conclusions:**

The implementation of strategies to reduce the prevalence and incidence of vision and hearing difficulties has the potential to increase the number of years spent without ADL/IADL limitations.

Both hearing and vision difficulties are some of the most common deficits experienced by older adults and increase in prevalence with age (1). Having lived most of their lives as fully sighted and hearing individuals, older adults, in particular, have difficulty adjusting their communication styles to other modalities (2). Therefore, the age-related decline in sensory function can negatively affect multiple aspects of an individual’s life, including their health and well-being (1,3,4).

Hearing difficulty is ranked as the third most common cause of years lived with disability (5) and is the most common sensory deficit in older people (6). It affects approximately one third of adults from 61 to 70 years of age and more than 80% of those older than 85 years. Age-related hearing difficulty has been associated with comorbidity, disability, and poor quality of life, affecting independent living and overall well-being (7), loneliness (8), as well as dementia and cognitive function (9,10). More specifically, associations have been shown with mobility limitations, difficulties with activities of daily living (ADLs) or instrumental activities of daily living (IADLs), and frailty (11–13).

Vision difficulty has been shown to be associated with a loss of balance and hence is among the leading risk factors for falls and fall-related injuries (14,15). Poor vision is an independent risk factor for physical and functional disability, as shown in various studies (16–18). A study in the United States found that older adults with moderate or severe visual difficulty have greater odds of incident frailty (19). In terms of psychosocial health, vision difficulty has been associated with depression and fewer social interactions (20,21). Vision difficulty is also associated with higher odds of depression, not only for the individual affected but also for their spouse, too (22).

Combined vision and hearing difficulties (hereafter defined as dual sensory difficulty) can cause even larger limitations in everyday activities than reporting only 1 difficulty, and it puts the individual at higher risk for negative health outcomes. The following issues have been found to be linked to dual sensory impairment: difficulty with ADLs or IADLs and functional dependence (1,16), communication impairment and social isolation, and depression (23).

Although indicators of health are important, it is becoming increasingly recognized that quantification of the quality of remaining years of life is also crucial (24). The concept of “health expectancy” is now widely used for this purpose as this is the expected number of remaining years of life spent in favorable states of health at a given age. Health expectancy indicators are useful in comparing the health of different populations, and they also have value for monitoring time trends and inequalities in population health because they combine data on both mortality and morbidity or disability (25). Health status indicators used to compute life expectancy range from objective measures of physiologic, disease, and functional status to subjective measures of self-perceived health (26). Nevertheless, the use of limitations with ADL/IADL is more widely accepted, especially in cross-country comparative studies (25), because these measures are less sensitive to cultural factors.

To date, however, only a few studies have identified the impact of hearing and vision difficulties on life and health expectancy, and they have mixed findings. For instance, Chen et al. (27) and Mathers et al. (28) showed that hearing and vision difficulties exhibited major influence in China and Australia, respectively. While Jagger et al. found that visual and hearing diseases were less influential disabling diseases compared to other conditions (29). However, none of those studies measured the impact of dual sensory difficulty on life expectancy without ADL/IADL limitations, and all used cross-sectional data to calculate life expectancy. This study aims to address this gap by estimating to what extent life expectancy without ADL/IADL limitations differs by the presence of hearing, vision, or dual sensory difficulty. We use two large nationally representative studies of aging in England and the United States to assess the extent to which sensory difficulty shows different associations with health expectancy in the two countries.

## Method

Detailed information about data harmonization and statistical methods is available in the Supplementary Material.

### Data

We used longitudinal data from the U.S. Health and Retirement Study (HRS) and the English Longitudinal Study of Ageing (ELSA). These are the 2 longest-running studies in the HRS family and have available data on mortality up to 2013. Established 10 years after HRS, ELSA was designed to be comparable in terms of population sampling, periodicity, and content of the survey (including the specific wording of questions) (30,31). The 2 studies have been described in detail elsewhere (32,33). Comparability between the 2 studies was maximized by using harmonized data files from 6 waves between 2002/2003 and 2012/2013 (a period for which mortality was available in both studies), available from The Gateway to Global Aging Data (g2aging.org), a data and information platform developed to facilitate cross-country analyses. In both cohorts, we included people who, in 2002/2003, were aged 50+ with valid data on health, vision, hearing, and wealth, resulting in analytical samples of 10 756 (out of the 11 391 ELSA members in 2002/2003) and 17 758 HRS members in 2002 aged 50 and over (refreshment samples added after 2002 are excluded from these analyses).

The sample size at the subsequent 5 waves was 8 453 in ELSA and 15 825 in HRS; 7 249 in ELSA and 14 308 in HRS; 6 388 in ELSA and 13 086 in HRS; 5 982 in ELSA and 11 588 in HRS; and 5 449 in ELSA and 10 520 in HRS.

### Measures

#### Outcome

At each wave in both studies, all participants were asked whether they had difficulties in performing ADL (eg, dressing, walking across a room, bathing or showering, eating, getting in/out of bed, using the toilet) and IADL (eg, using a map, preparing a hot meal, shopping for groceries, making phone calls, taking medications, managing money). Responses were summed and categorized as not having limitations (0 or 1 ADL/IADL) and having limitations (2+ ADL/IADL). The cutoff of 2 or more ADL or IADL was chosen based on the average number of ADL or IADL limitations reported by people (in ELSA) who, at baseline, received health or disability benefits (34). Health expectancy based on ADL/IADL limitations is named here as life expectancy without ADL/IADL limitations (LEWL).

*Mortality* up to March 2013 was ascertained from linked register data for ELSA and through linkages to the National Death Index and reports from survivors for HRS. By the end of the follow-up, 5 635 deaths occurred in the U.S. sample and 2 471 in the English sample.

#### Vision and hearing difficulties

During the main interview, people were asked the following questions: “Is your eyesight (using glasses or corrective lens if you use them) …” and “Is your hearing (using a hearing aid if you use one) …” Responses were rated on a scale of 5 from excellent to poor. People were defined as having difficulties with vision or hearing if they rated their vision or hearing as fair or poor (including if they were using glasses and hearing aids) (35). Dual sensory difficulty was defined as having both vision and hearing difficulties at baseline.

*Age* was measured in years (range 50–100). *Sex* was coded as Male and Female. We used *total household wealth* as an indicator of socioeconomic status, defined as the sum of net financial wealth and net housing wealth less all debts (36). The continuous variable was divided into 3 groups (ie, each containing 33% of the sample).

### Statistical Analyses

The total length of time in the study was 10 years (from 2002/2003 to 2012/2013, average follow-up of 6 years).

To estimate total life expectancy and life expectancy without ADL/IADL limitations from the ages of 50 to 100 from repeated measures, we used multistate life table models (25,37). Supplementary Material provides a detailed description of the methodology used to compute healthy life expectancies.

We used the Stochastic Population Analysis for Complex Events program (37) in SAS 9.2 (SAS Institute, Cary, NC) to estimate multistate life table functions. This program has 2 main components: the data component, which prepares the input data sets, and the statistical component, in which transition probabilities and the multistate life table functions and their variances are estimated. We defined the following 3 health states: healthy (without ADL/IADL limitations), unhealthy (with ADL/IADL limitations), and dead. There were 4 possible transitions between the health states, namely: healthy to unhealthy (onset), unhealthy to healthy (recovery), healthy to dead, and unhealthy to dead. During the statistical component, age-specific transition probabilities for all possible transitions are estimated from the data using multinomial logistic regression with age, sex, wealth tertiles, vision, and hearing difficulties, and the interaction term between age and vision and hearing difficulties. LEWL estimates for ages 50+ are then calculated based on these estimated transition probabilities using a stochastic (microsimulation) approach. By using microsimulation, it is possible to simulate the life paths of the members of the population in order to derive several summary statistics of the population dynamics. For each study separately, the program generated individual trajectories for a simulated cohort of 100 000 persons with distributions of covariates at the starting point based on the observed study-specific prevalence by 5-year age group and sex. Analyses were run with sensory difficulty measured at baseline (dual sensory difficulty, hearing difficulty, vision difficulty, and neither). Variability for these multistate life table estimates (variances, standard errors, and corresponding 95% confidence intervals) was computed using a bootstrap method with 500 replicates for the whole analysis process (multinomial analysis and simulation steps). The method deals with unevenly spaced observations.

### Sensitivity Analysis

To investigate the possibility of reverse causation, we ran estimates for people who were healthy at baseline (HRS *N* = 11 640 and *N* = 7 369 ELSA).

## Results

The characteristics of the study cohorts at baseline (2002) are presented in Table 1. The average age of men and women in England was 65 and that of men and women in the United States was 69. The prevalence of disability was 13% among men in both England and the United States, although it was higher among women (16% in England and 19% in the United States).

**Table 1:** Baseline Sample Characteristics, by Gender and Cohort Study, England and United States 2002

	ELSA		HRS	
	Men	Women	Men	Women
Sample size	4 981	5 775	7 388	10 370
Age mean (*SE*)	64.9 (9.9)	65.3 (10.4)	68.8 (9.15)	69.0 (10.5)
Wealth tertiles (%)				
High	32.8	29.2	37.6	30.3
Middle	32.8	33.1	33.8	33.0
Low	34.4	29.2	28.7	36.7
2+ ADL/IADL limitations (%)	12.9	15.5	13.2	19.2
Dual sensory difficulty (%)	6.5	5.2	9.5	6.7
Hearing difficulty (%)	20.8	11.5	18.0	8.3
Vision difficulty (%)	7.8	11.6	9.7	14.0
Neither (%)	64.9	71.7	62.8	71.0

*Notes*: Percentages and mean are estimated using sampling weights. ADL = activity of daily living; ELSA = English Longitudinal Study of Ageing; HRS = Health and Retirement Study; IADL = instrumental activity of daily living; *SE* = standard error.

At baseline, over 62% of men and 71% of women in both cohorts reported no sensory difficulties. One in 5 men in England reported hearing difficulty. This proportion is slightly lower in the United States (18%). The proportions of women reporting hearing difficulty in England and the United States were 12% and 8%, respectively. In England, the prevalence of people reporting vision difficulty was 7.8% in men and 11.6% in women, whereas, in the United States, it was 9.7% and 14%, respectively. In England, 7% of men and 5% of women reported dual sensory difficulty (ie, both hearing and vision difficulties), slightly less than men and women in the United States (10% and 7%, respectively).

Total life expectancy estimates at the ages of 50, 60, and 70, by gender and country, according to vision and hearing difficulties, are reported in Table 2. The estimates represent the total average number of years (sum of healthy and unhealthy remaining years of life) that people can expect to live at any given age, according to whether they have vision and hearing difficulties. For example, at age 50, men in the United States with dual sensory difficulty could expect to live 25.8 more years and women 29.1 compared to 32.3 of men and 35.8 of women with neither vision nor hearing difficulties. The gap between those with dual sensory difficulty and those with neither was slightly lower in England than in the United States. At the age of 70, the difference in total life expectancy between people with dual sensory difficulty and people with neither was 3–4 years.

**Table 2:** Estimates of Total Life Expectancy at the Age of 50 According to Vision and Hearing Difficulties, by Gender and Cohort Study England and Unites States 2002–2013

	ELSA		HRS	
	Men	Women	Men	Women
	Age 50	Age 50	Age 50	Age 50
Dual sensory difficulty	26.9 (25.4; 28.7)	31.3 (29.8; 33.3)	25.8 (24.4; 27.9)	29.1 (27.8; 30.9)
Hearing difficulty	30.9 (30.0; 31.9)	34.8 (33.8; 35.6)	32.2 (31.3; 33.6)	34.7 (33.3; 35.8)
Vision difficulty	28.9 (27.2; 30.8)	32.8 (31.6; 34.3)	24.6 (23.3; 27.4)	29.9 (28.8; 31.5)
Neither	32.3 (31.9; 32.8)	36.0 (35.5; 36.3)	32.3 (31.5; 32.9)	35.8 (35.3; 36.3)
	Age 60	Age 60	Age 60	Age 60
Dual sensory difficulty	19.5 (17.9;20.4)	23.3 (22.0;24.8)	19.1 (18.0;20.1)	21.4 (20.3;22.2)
Hearing difficulty	22.1 (21.2;22.8)	25.8 (24.5;26.2)	23.1 (22.2;23.7)	25.1 (24.6;26.1)
Vision difficulty	19.6 (18.5;21.1)	24.3 (23.2;25.3)	18.5 (17.4;19.3)	21.1 (20.4;22.0)
Neither	23.3 (22.9;23.7)	26.9 (26.5;27.3)	23.9 (23.3;24.3)	26.4 (26.0;26.9)
	Age 70	Age 70	Age 70	Age 70
Dual sensory difficulty	12.4 (11.8;13.0)	14.8 (14.0;15.8)	12.7 (12.1;13.4)	14.3 (13.6;15.2)
Hearing difficulty	14.0 (13.2;14.5)	16.9 (16.3;17.6)	15.4 (14.9;16.0)	17.3 (16.6;17.8)
Vision difficulty	12.7 (12.1;13.6)	15.7 (15.0;16.5)	12.7 (11.9;13.2)	15.0 (14.4;15.8)
Neither	14.9 (14.5;15.3)	17.9 (17.5;18.2)	16.0 (15.7;16.4)	18.3 (17.9;18.6)

*Notes*: Estimates from models with covariates age, sex, and wealth and interaction term between age and vision and hearing difficulties. ELSA = English Longitudinal Study of Ageing; HRS = Health and Retirement Study.

**Figure 1.**
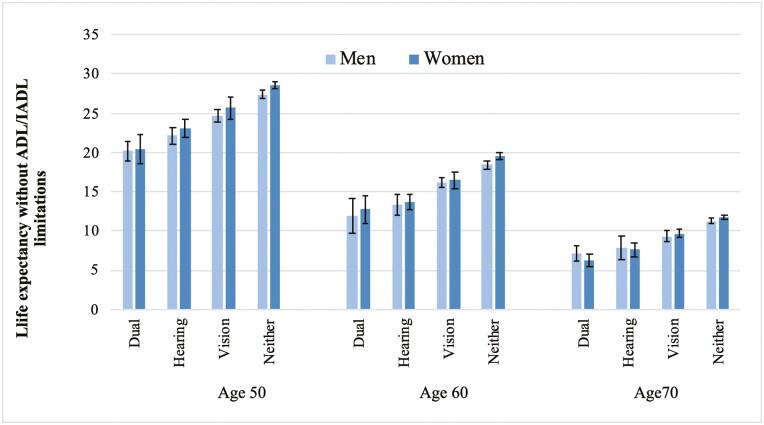
Estimates of life expectancy without activities of daily living (ADL)/instrumental activities of daily living (IADL) limitations (in years) according to vision and hearing difficulties, by gender and age, England 2002–2013. Estimates from models with covariates age, sex, and wealth and interaction term between age and vision and hearing difficulties.

In Figure 1, we report estimates of years expected to live without ADL/IADL limitations (LEWL) at the age of 50, 60, and 70 for men and women in England according to vision and hearing difficulties (in Supplementary Table 1, we provide the data for this figure). At the age of 50, men and women in England with neither vision nor hearing difficulties could expect to live 27 and 29 additional years without ADL/IADL limitations compared to 20 years for men and women reporting both difficulties. Men and women reporting hearing difficulty at the age of 50 could expect to live 22–23 additional years without ADL/IADL limitations, whereas those with vision difficulty 25–26 additional years. By the ages of 60 and 70, the estimates become closer.

**Figure 2.**
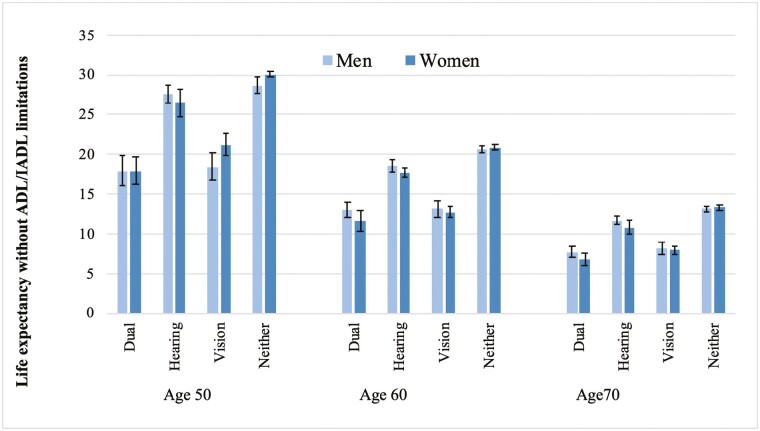
Estimates of life expectancy without activities of daily living (ADL)/instrumental activities of daily living (IADL) limitations (in years) according to vision and hearing difficulties, by gender and age, United States 2002–2013. Estimates from models with covariates age, sex, and wealth and interaction term between age and vision and hearing difficulties.

Results for men and women in the United States are reported in Figure 2 (in Supplementary Table 1, we provide the data for this figure). We observed a similar trend to that of England: People with both vision and hearing difficulties can expect to live up to 12 fewer years without ADL/IADL limitations than those with neither. However, different from what we observed in England, people with vision difficulty were the least likely to live longer without ADL/IADL limitations.

### Sensitivity Analysis

In Supplementary Table 2, we report the results of the sensitivity analysis restricted to those individuals who were healthy at baseline (without ADL/IADL limitations). Differences in total life expectancy by vision and hearing difficulties are similar to those reported in Table 2 of the main analyses. Similar results were also obtained for LEWL (Supplementary Tables 1 and 2), which suggest that estimates are unlikely to be biased by health selection. We observed a reduction in the difference in the estimates of LEWL between men and women in the United States with hearing difficulty.

## Discussion

Our findings, from 2 large nationally representative studies of aging in the United States and England, showed that self-reported single (hearing or vision only) and dual (both hearing and vision) difficulties in older adults were associated with shorter life expectancy as well as shorter life expectancy without ADL/IADL limitations. Similarly, people with vision, hearing, or dual sensory difficulties live more years with ADL/IADL limitations than those with no difficulties. The association is greater among those with dual sensory difficulty in both countries. The gap in the years expected to live without ADL/IADL limitations between those reporting no difficulties and those reporting dual sensory difficulty was as large as 12 years. This finding is supported by a study using data from the Panel on Health and Ageing of Singaporean Elderly, which found that older adults with dual sensory difficulty had fewer years without limitation in physical function and in ADLs than those without difficulty (38). Individuals with single sensory difficulty (hearing or vision only) are more likely to use the other normally functioning sensory to compensate for their impairment (3). The concurrent vision and hearing difficulties may remove those compensatory mechanisms and lead to greater dependence. For instance, poorer vision will limit individuals with hearing impairment from seeing the sign language interpreter. A qualitative study identified that, in addition to difficulties with communication, individuals with dual sensory difficulty found it challenging to navigate unfamiliar surroundings, leading to a loss of independence (39).

We further found that at the ages of 50 and 60, among English men and women, self-reported hearing difficulty was associated with fewer years lived without ADL/IADL limitations than vision difficulty. In contrast, in the United States, self-reported vision difficulty led to fewer years lived without ADL/IADL limitations than hearing difficulty. It is not entirely clear why that would be the case. It is possible that in the United States, as identified in a review examining the impact of sensory difficulty on life expectancy and health-adjusted life expectancy (40), the impact of visual difficulty on health expectancy reduction is greater for visual difficulty than for hearing difficulty in those studies. For instance, one of the studies in the review found that the life expectancy with disability among men and women in China caused by diseases of the eye and adnexa was longer than that by diseases of the ear and mastoid process (27). Hearing and visual difficulties may affect older people differently. A study in Germany found that the difficulties in maintaining social relationships among older people with hearing difficulty were mainly due to the challenges in using the phone and the presence of communication problems (41). In comparison, those with visual difficulty reported problems writing cards or letters and leaving the house, restricting them from meeting other people in person. Social isolation may mediate the detrimental impact of hearing and visual difficulties on physical functioning, as the associations between social isolation and poorer physical functioning have been demonstrated in previous work (42,43).

Hearing and visual difficulties could also be associated with a higher risk of disabilities through mechanistic pathways involving their effects on cognitive load, mental health, and life satisfaction. In the cognitive load on perception hypothesis, the sensory difficulty may cause an increase in the cognitive resources required for processing the degraded auditory signals, which may lead to a higher burden on cognition (44). Hearing and visual difficulties are associated with a higher risk of depression and less life satisfaction (45), which may lead to higher estimates of health expectancy (46). Finally, the common-cause hypothesis, in which hearing and visual difficulties and physical and cognitive function declines may be caused by a common underlying pathology, such as cardiometabolic diseases and biological aging, should also be considered (47,48). Further studies will be needed to clarify this association, as understanding these potential mechanisms will be crucial for informing interventions to mitigate hearing and visual difficulties as well as reduce the risk of disabilities.

At the age of 50, women in England with hearing, visual, or both were expected to live longer without ADL/IADL limitations than men. Prior studies reported that women with hearing or vision difficulties have longer life expectancy with disabilities than men with the same difficulties (38,49). It reflected the “gender paradox,” which states that women live longer than men but spend a longer duration of life with disability and poor health (26,50). The similar LEWL reported in this study at the age of 60 or older between men and women may reflect the similar access to health care interventions to improve sensory function for both genders in England and the United States. A study using data from the largest pharmacy-led health and beauty retailers in the United Kingdom revealed that gender did not significantly predict hearing aid adoption (51).

The main strengths of our study are the use of large representative data from well-known longitudinal studies of aging, designed to be representative. We used objective measures of mortality in both studies and rich data collected over time. The results of our study should be considered in light of some limitations. Although objective measures of hearing and vision difficulties would have been preferable, self-reported measures of sensory function are commonly used in prior epidemiological studies (9,10). Self-reported visual (35) and hearing functions (52) have been validated as a suitable indicator of sensory difficulty in older people in Ireland. Another possible limitation is that different results could be obtained when adopting different measures of health expectancy. We opted for limitations with ADL and IADL because it is widely accepted and recommended in international comparisons. However, measures of chronic morbidity, self-rated health, mobility, or disability could also be used and results might differ from those reported here. A further limitation is that participants in longitudinal studies tend to be healthier than those in the general population. It is possible that the sample was affected by mortality selection, which indicates that when mortality at younger ages is high, it affects frail people first; therefore, survivors at older ages are a selected group of healthier people. We compared estimates of total life expectancy obtained from our data with national life tables and found similar results for England and slightly higher estimates for the United States compared to life tables (53,54). Thus, it is unlikely that we have overestimated life expectancy and life expectancy without ADL/IADL limitations.

In conclusion, our study found that, among adults aged 50 and older living in England and the United States, sensory difficulty is associated with both reduced life expectancy and life expectancy without ADL/IADL limitations, and the impact is greater among those with dual sensory difficulty. It implies that sensory difficulty should not be considered a normal aspect of the aging process. Despite being highly prevalent, age-related vision and hearing difficulties are often underreported, and their impact is poorly recognized (55,56), which may explain the low rates of interventions to improve the conditions, including hearing aid use (57). This study thus highlights the need for greater recognition by health policy-makers of the importance of sensory difficulty as a public health issue and of the potential benefits from early screening, management, and rehabilitation of hearing and vision difficulties in older adults.

## Supplementary Material

glad136_suppl_Supplementary_Material

## Data Availability

The data used in this work can be obtained free upon registration at https://g2aging.org/; codes to compute healthy life expectancy can be downloaded at http://sites.utexas.edu/space/
