# Supplementary material for: Vision and Hearing Difficulties and Life Expectancy Without ADL/IADL Limitations: Evidence From the English Longitudinal Study of Ageing and the Health and Retirement Study
Source: J Gerontol A Biol Sci Med Sci. 2023 May 26;79(2):glad136. doi: 10.1093/gerona/glad136 (PMC10799758; doi:10.1093/gerona/glad136)
Supplement: glad136_suppl_Supplementary_Material [file glad136_suppl_supplementary_material.docx]

**Supplementary Materials**

**1. Harmonization of the measures used**

For the analyses of this paper we used ELSA and HRS harmonized data available from The Gateway to Global Aging Data (g2aging.org). The Gateway to Global Aging Data is a project sponsored by the National Institute on Aging, to facilitate cross-national comparative studies on aging using a family of health and retirement studies around the world. Harmonized datasets are developed as a single file which includes all longitudinal waves of the study of interest. Harmonized variables use a consistent and intuitive variable naming convention across surveys to identify directly comparable measures between studies. Each Harmonized data file has its own codebook which carefully explains how each harmonized variable was created and highlights any differences between the same variable in different studies. The HRS harmonized data documentation is available at

<http://hrsonline.isr.umich.edu/modules/meta/rand/randhrsp/randhrs_P.pdf>

And the ELSA harmonized data documentation is available at

<http://doc.ukdataservice.ac.uk/doc/5050/mrdoc/pdf/5050_harmonized_elsa_e.pdf>

***Activities of daily living and Instrumental activities of daily living***

In both studies difficulties with activities of daily living (bathing, eating, dressing, using the toilet, walking across a room, and getting in or out of bed) and instrumental activities of daily living (using a telephone, taking medication, handling money, shopping, preparing meals) are asked to respondents at each wave and include the same response categories.

***Self-reported hearing and vision impairment***

In both studies people were asked to rate their vision and hearing on a scale of 5 from excellent to poor. People were defined as having vision or hearing impairment if they rated their vision or hearing as fair or poor. Dual impairment was defined as having both vision and hearing impairment.

***Socioeconomic indicators***

HRS and ELSA both contain high-quality wealth modules using a comprehensive and detailed set of questions on the components of wealth.^3^ Total family net non-pension wealth in both studies is defined as the sum of financial wealth, physical wealth (such as business wealth, land, or jewels), and housing wealth (primary and secondary residential housing wealth), minus all debts. For more details the readers can refer to section C of the HRS harmonized documentation file (page 854) and Section E of the ELSA harmonized documentation file (page 297). The continuous variable of wealth within each study was recoded into equally sized tertiles.

**2 Computation of healthy life expectancy**

The computation of health expectancy using the Sullivan method is usually applied to cross-sectional data, and requires life tables and information on age specific proportions of the population in healthy or unhealthy stages. These proportions are prevalence measures of the actual and current health status of a real population and are used to divide years lived in the life table population. With panel data reliable estimates of life table inputs cannot be obtained.

The multi-state life table (MSLT) model has been developed to analyse stochastic processes that involve multiple and recurrent events (typical of longitudinal data), in order to estimate expected duration in various states. The MSLT method uses a set of transition probabilities from

healthy, unhealthy and to death estimated using longitudinal data.

We defined the following three health states: healthy (without ADL/IADL-limitations), unhealthy (with ADL/IADL-limitations), and dead. There were four possible transitions between the states, namely: healthy to unhealthy (onset), unhealthy to healthy

(recovery), healthy to dead, unhealthy to dead.

The advantages of multistate life table methods are: it is based on incidence measures representing current health transitions; it allows movement in both directions between all surviving health states; it allows death rates to differ by health state so it takes into account the different mortality profiles by health status. The estimation of transition schedules is very important and can be done using logistic regression, multinomial logistic regression, or proportional hazards regression.

We used the Stochastic Population Analysis for Complex Events (SPACE) program in SAS 9.2 to

estimate MSLT functions. There are two main components to this program: the data component,

which prepares the input datasets, and the statistical component in which transition probabilities and

the multistate life table functions and their variances are estimated. Specifically, during the statistical

component age-specific transition probabilities for all possible transitions are estimated from the data

using multinomial logistic regression conditional on age, sex and sensory impairment (included as

covariate in the model). Health expectancies for ages 50+ are then calculated based on these

estimated transition probabilities using a stochastic (micro-simulation) approach. By using microsimulation it is possible to simulate the life paths of the members of the population in order to derive several summary statistics of the population dynamics. For each study separately, the program

generated individual trajectories for a simulated cohort of 100,000 persons with distributions of

covariates at the starting point based on the observed study-specific prevalence by five year age group

and sex. Multinomial logistic regressions were adjusted for wealth tertiles. Variability for these multistate life table estimates (variances, standard errors and corresponding 95% confidence intervals) were computed using a bootstrap method with 500 replicates for the whole analysis process (multinomial analysis and simulation steps).

More information can be found at <http://www.cdc.gov/nchs/data_access/space.htm>.

The SPACE manual is available at <ftp://ftp.cdc.gov/pub/Health_Statistics/NCHS/Software/space/SPACE_manual.pdf>

The annotated codes can be found at

<ftp://ftp.cdc.gov/pub/Health_Statistics/NCHS/Software/space/sas/MSLT_RAD2COV_S.pdf>

<ftp://ftp.cdc.gov/pub/Health_Statistics/NCHS/Software/space/sas/MSLT_SIMxCOV_S.pdf>

ftp://ftp.cdc.gov/pub/Health_Statistics/NCHS/Software/space/sas/MSLT_SIMxCOV_M_DX.pdf

<ftp://ftp.cdc.gov/pub/Health_Statistics/NCHS/Software/space/sas/SMPEM_SIMDUR.pdf>

<ftp://ftp.cdc.gov/pub/Health_Statistics/NCHS/Software/space/sas/SMPEM_SIMxCOV_S.pdf>

**Supplementary Table 1** **Estimates of life expectancy without ADL/IADL-limitations (in years) according to vision and hearing difficulties, by gender and age, England and the US 2002-2013**

|  | **ELSA** | |  | **HRS** | |  |
| --- | --- | --- | --- | --- | --- | --- |
|  | **Men** | **Women** |  | **Men** | **Women** |  |
|  | **Age 50** | **Age 50** |  | **Age 50** | **Age 50** |  |
| Dual sensory difficulty | 20.2  (18.9; 22.2) | 20.5  (16.2; 20.7) | 17.9  (16.0; 20.9) | | 18.0  (16.2; 20.7) |  |
| Hearing difficulty | 22.1  (21.1; 23.5) | 23.0  (24.7; 28.3) | 27.6  (26.4; 29.0) | | 26.5  (24.7; 28.3) |  |
| Vision difficulty | 24.6  (23.8; 25.5) | 25.7  (19.8; 22.6) | 18.5  (16.8; 21.6) | | 21.2  (19.8; 22.6) |  |
| Neither | 27.4  (26.9; 27.9) | 28.5  (29.7; 30.8) | 28.7  (27.7; 29.4) | | 30.1  (29.7; 30.8) |  |
|  | **Age 60** | **Age 60** | **Age 60** | | **Age 60** |  |
| Dual sensory difficulty | 11.9  (9.7; 13.1) | 12.8  (10.3; 12.8) | 13.0  (12.0; 13.9) | | 11.6  (10.3; 12.8) |  |
| Hearing difficulty | 13.4  (12.1; 14.5) | 13.8  (17.0; 18.7) | 18.6  (17.9; 19.2) | | 17.7  (17.0; 18.7) |  |
| Vision difficulty | 16.3  (15.6; 17.0) | 16.5  (12.1; 13.5) | 13.1  (12.0; 13.8) | | 12.7  (12.1; 13.5) |  |
| Neither | 18.4  (17.9; 18.9) | 19.5  (20.5; 21.3) | 20.7  (20.2; 21.1) | | 20.8  (20.5; 21.3) |  |
|  | **Age 70** | **Age 70** | **Age 70** | | **Age 70** |  |
| Dual sensory difficulty | 7.1  (6.1; 8.0) | 6.3  (6.0; 7.6) | 7.7  (7.0; 8.6) | | 6.8  (6.0; 7.6) |  |
| Hearing difficulty | 7.8  (6.3; 8.4) | 7.6  (9.9; 11.8) | 11.7  (11.1; 12.2) | | 10.8  (9.9; 11.8) |  |
| Vision difficulty | 9.3  (8.6; 9.9) | 9.7  (7.3; 8.7) | 8.2  (7.4; 8.7) | | 7.9  (7.3; 8.7) |  |
| Neither | 11.3  (10.9; 11.7) | 11.8  (13.0; 13.7) | 13.1  (12.8; 13.6) | | 13.3  (13.0; 13.7) |  |

Estimates from models with covariates age, sex, and wealth and interaction term between age and vision and hearing difficulties.

**Supplementary Table 2 Estimates of total life expectancy and life expectancy without ADL/IADL-limitations at the age of 50 according to vision and hearing difficulties, by gender and cohort study among healthy individuals at baseline, England and Unites States 2002-2013**

|  | **ELSA** | |  | **HRS** | |  |
| --- | --- | --- | --- | --- | --- | --- |
|  | **Men** | **Women** |  | **Men** | **Women** |  |
| **Life Expectancy** | **Age 50** | **Age 50** |  | **Age 50** | **Age 50** |  |
| Dual sensory difficulty | 27.3  (25.9; 29.1) | 32.2  (30.6; 34.1) | 26.3  (24.5; 29.4) | | 29.8  (28.6; 31.5) |  |
| Hearing difficulty | 31.2  (30.3; 32.1) | 35.0  (34.0; 35.7) | 32.3  (31.3; 33.9) | | 35.0  (33.5; 36.0) |  |
| Vision difficulty | 29.3  (28.1; 31.6) | 33.2  (32.3; 34.6) | 25.4  (24.0; 27.4) | | 30.4  (29.2; 31.7) |  |
| Neither | 32.4  (32.0; 32.9) | 36.1  (35.6; 36.4) | 32.6  (31.7; 33.1) | | 35.9  (35.4; 36.4) |  |
| **Life expectancy without ADL/IADL-limitations** | | |  | |  |  |
| Dual sensory difficulty | 20.8  (19.1; 22.2) | 22.3  (20.8; 24.4) | 19.9  (18.0; 23.7) | | 20.0  (18.6; 22.3) |  |
| Hearing difficulty | 26.3  (25.5; 27.1) | 27.5  (26.7; 28.6) | 28.0  (26.6; 29.6) | | 27.3  (25.5; 28.7) |  |
| Vision difficulty | 23.9  (22.2; 25.7) | 24.9  (23.6; 26.1) | 20.0  (18.7; 21.6) | | 22.2  (20.9; 23.2) |  |
| Neither | 29.0  (28.5; 29.3) | 30.7  (30.2; 31.1) | 29.3  (28.4; 29.8) | | 30.3  (29.8; 30.9) |  |

Estimates from models with covariates age, sex, and wealth and interaction term between age and vision and hearing difficulties. HRS N=11,640 and N= 7,369 ELSA
